# Nucleo-cytoplasmic transport as a therapeutic target of cancer

**DOI:** 10.1186/s13045-014-0085-1

**Published:** 2014-12-05

**Authors:** Giovanni Luca Gravina, William Senapedis, Dilara McCauley, Erkan Baloglu, Sharon Shacham, Claudio Festuccia

**Affiliations:** Department of Biotechnological and Applied Clinical Sciences, University of L’Aquila, L’Aquila, Italy; Karyopharm Therapeutics, Inc., 85 Wells Avenue, Newton, MA USA

**Keywords:** Exportin-1 (XPO1), Chromosome Region Maintenance 1 (CRM1), Nuclear-cytoplasmic transport, Cancer treatment

## Abstract

Shuttling of specific proteins out of the nucleus is essential for the regulation of the cell cycle and proliferation of both normal and malignant tissues. Dysregulation of this fundamental process may affect many other important cellular processes such as tumor growth, inflammatory response, cell cycle, and apoptosis. It is known that XPO1 (Exportin-1/Chromosome Region Maintenance 1/CRM1) is the main mediator of nuclear export in many cell types. Nuclear proteins exported to the cytoplasm by XPO1 include the drug targets topoisomerase IIα (topo IIα) and BCR-ABL and tumor suppressor proteins such as Rb, APC, p53, p21, and p27. XPO1 can mediate cell proliferation through several pathways: (i) the sub-cellular localization of NES-containing oncogenes and tumor suppressor proteins, (ii) the control of the mitotic apparatus and chromosome segregation, and (iii) the maintenance of nuclear and chromosomal structures. The XPO1 protein is elevated in ovarian carcinoma, glioma, osteosarcoma, pancreatic and cervical cancer. There is a growing body of research indicating that XPO1 may have an important role as a prognostic marker in solid tumors. Because of this, nuclear export inhibition through XPO1 is a potential target for therapeutic intervention in many cancers. The best understood XPO1 inhibitors are the small molecule nuclear export inhibitors (NEIs; Leptomycin B and derivatives, ratjadones, PKF050-638, valtrate, ACA, CBS9106, selinexor/KPT-330, and verdinexor/KPT-335). Selinexor and verdinexor are orally bioavailable, highly potent, small molecules that are classified as Selective Inhibitors of Nuclear Export (SINE). KPT-330 is the only NEI currently in Phase I/II human clinical trials in hematological and solid cancers. Of all the potential targets in nuclear cytoplasmic transport, the nuclear export receptor XPO1 remains the best understood and most advanced therapeutic target for the treatment of cancer.

**Electronic supplementary material:**

The online version of this article (doi:10.1186/s13045-014-0085-1) contains supplementary material, which is available to authorized users.

## Introduction

Export of mRNA and specific proteins from the nucleus is a key step in intracellular signaling and can lead to cell proliferation or apoptosis [1]. Cancer cells utilize the processes of nuclear-cytoplasmic transport through the nuclear pore complex to stimulate tumor growth and to effectively evade apoptotic mechanisms [1]. It is known that XPO1 (Exportin-1/Chromosome Region Maintenance 1/CRM1) is the main mediator of nuclear export in many cell types. XPO1 interacts with nucleoporins (NUP214 and NUP88) in the nuclear pore complex [2] and transports cargo proteins containing nuclear export signals (NES) out of the cell nucleus [3]. NES are short leucine-rich sequences that can be found in many shuttling proteins, including numerous tumor suppressors and oncogenes [3] (see NESdb database; http://www4.utsouthwestern.edu/chooklab/resources.htm). XPO1 protein mediates cell proliferation through several pathways: (i) the sub-cellular localization of NES-containing oncogenes and tumors suppressor proteins, (ii) the control of the mitotic apparatus and chromosome segregation, and (iii) the maintenance of nuclear and chromosomal structures.

The level of XPO1 protein remains constant throughout the cell cycle [4] and is mainly localized to the nuclear envelope in highly specialized cellular bodies called CRM1 nucleolar bodies (CNoBs) [5],[6]. CNoBs depend on RNA polymerase I activity, indicating a role in ribosome biogenesis [7]. Shuttling of specific proteins out of the nucleus is essential for the regulation of cell cycle and proliferation of both normal and malignant tissues [8]-[11]. Examples of nuclear effectors which are exported into the cytoplasm in cancer include the drug targets topoisomerase IIα [12] and BCR-ABL [13] and tumor suppressor proteins such as Rb [14], APC [15], p53 [16], p21 [17], and p27 [18] (reviewed in Table 1). This makes nuclear export inhibition a potential target for therapeutic intervention in cancer [19],[20].

**Table 1:** **Molecular consequences associated with XPO1 inhibition**

Target (nuclear accumulation)	Biological effects	References
Cyclin D1	Protein degradation, reduction of cell proliferation and increased apoptosis	[17],[31]
p21	Reduction of cell proliferation	[17]
p27	Reduction of cell proliferation	[18],[34]
p53	Restoration of nuclear p53 and p53-mediated response to stress	[16],[30],[33],[59]
FOXO proteins	Activates the transcription of genes that promote cell cycle arrest, apoptosis and down-modulate Wnt/β-catenin signals	[30],[34]-[39]
IκB	Attenuates constitutively activated NF-κB and causes apoptosis in cancer cells	[40]-[42]
BRCA1	Resistance versus PARP inhibitors	[43]-[45]
Survivin	Increased apoptosis	[46]-[51]
Fbw7	Degrades nuclear Notch-1 leading to decreased tumor promoting markers such as C-Myc, Cyclin-D1, Hes1 and VEGF.	[52]
Topo IIα	Sensitization to Topoisomerase II poisons	[53]
Nucleophosmin	Once within the nucleus it could, in principle drive Bax translocation.	[54]-[56]
FAS activation	Activation of intrinsic apoptosis pathway	[57],[58],[60],[61]

### Prognostic role of XPO1 in solid tumors

The XPO1 protein is elevated in ovarian carcinoma, glioma, osteosarcoma, pancreatic, cervical and gastric cancers and may have an important role as prognostic marker in solid and hematologic tumors [11],[21]-[28]. XPO1 protein expression is increased in osteosarcoma when compared to non-tumor tissue [23]. High serum levels of alkaline phosphatase (ALP) are associated with increased expression of XPO1. From a clinical point of view, elevated expression of XPO1 is associated with increased tumor size and negative histological grade. High XPO1 protein expression is correlated with both poor progression-free (PFS) and overall survival (OS) in human osteosarcoma.

In ovarian [21] and cervical cancer [25], increased XPO1 nuclear and cytoplasmic protein expression was observed in malignant tissues when compared to benign lesions. XPO1 protein was also found differentially expressed in borderline tumors with respect to malignant ovarian cancer [21]. These findings suggest a continuum of expression from benign to malignant lesions encompassing more favorable outcomes for cervical cancer patients [25]. Cytoplasmic XPO1 protein expression was correlated with increased mitotic index, more aggressive tumor growth, advanced tumor stage, and poor OS [25]. XPO1 was shown to export COX-2 from the nucleus [29]. Jang and colleagues suggested that elevated expression of the XPO1 protein may cause COX-2 up-regulation [29]. In cervical cancer cell lines, silencing of the XPO1 protein by RNA interference resulted in increased cell death. This effect was found to be correlated with nuclear retention of p53 [16],[29],[30].

In patients suffering from Stage I and II pancreatic cancer, increased XPO1 protein expression was detected in malignant tissues [24]. Serum CEA and CA19.9 levels, two well-known prognostic markers in pancreatic cancer, correlated with increased XPO1 protein expression in human tissues. In addition, tumor size and presence of distant metastasis also correlated with increased levels of XPO1 protein. Therefore, it may be possible to use high XPO1 expression as a clinical parameter for predicting poor PFS and OS in pancreatic cancer. High XPO1 protein expression was significantly associated with high expression of phospho-serine10-p27, but reduced abundance of p27. Increased XPO1 led to increased cytoplasmic localization and degradation of p27, while phospho-serine10-p27 was resistant to XPO1-mediated nuclear export. Considered together, these results provide direct evidence of XPO1 nuclear export of p27 in pancreatic cancer.

A prognostic role of the XPO1 protein was established in gastric cancer (GC) [28]. A higher XPO1 expression rate (57.8%) was found in tissue derived from malignant lesions when compared to adjacent noncancerous tissues (6.7%). XPO1 protein expression was correlated with increased serum level of CEA, more advanced tumor stages, positive Her2 status, and distant metastasis. Using multivariate analysis, it was determined that high XPO1 expression was an independent indicator for GC survival.

### Molecular signals associated with XPO1 inhibition

**Figure 1:**
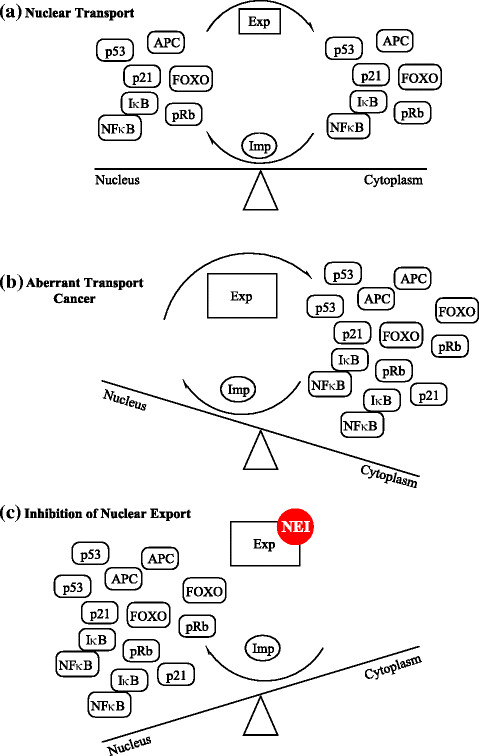
**Nucleo-cytoplasmic transport as therapeutic target in cancer.** The balance of nuclear transport in **(a)** normal and **(b)** cancer cells. **(c)** Inhibition of nuclear export by NEIs in cancer cells.

The physical separation of the genome from the cytoplasm by the nuclear envelope (NE) is a hallmark of the eukaryotic cell requiring the transport of macromolecules across the nuclear membrane to mediate their normal functions (Figure 1a). It is known that intracellular localization is deregulated in cancer [10],[11] (Figure 1b). Targeting nucleo-cytoplasmic transport could restore normal localization and function of tumor suppressor and oncoproteins (Figure 1c). The targeting of XPO1 by nuclear export inhibitors (NEI) induces apoptosis in cancer cell lines and slows tumor growth in xenograft mouse models. There are many different mechanisms which achieve this in cancer cells [16]-[18],[31]-[61]. These mechanisms are highlighted in Table 1.

### Small molecule nuclear export inhibitors (NEIs) and anti-cancer activity

**Figure 2:**
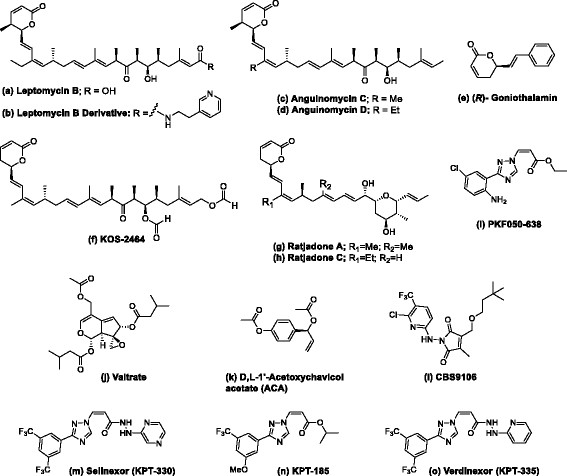
**Structures of Nuclear Export Inhibitors (NEIs).**

Of all the potential targets in nuclear-cytoplasmic transport, the nuclear export receptor XPO1 remains the most promising therapeutic target. Figure 2 summarizes some of the small molecule nuclear export inhibitors (NEIs) described in this review. Leptomycin B (LMB; Figure 2a) is the first specific NEI discovered [62]. LMB is a small molecule capable of disrupting protein-protein interactions that are typically difficult to target. When therapeutically evaluated in a single Phase I clinical trial in humans, LMB was found to exhibit severe dose-limiting toxicity, resulting in profound anorexia and malaise, which are potentially off-target effects [63]. In attempts to reduce the potential off-target effects of LMB, different derivatives were developed with improved pharmacological properties (Figure 2b) [64]-[72]. Several different natural products as well as semi-synthetic and synthetic compounds were identified, although they have not been tested in humans.

The long LMB lactone polyketide almost fills up the NES groove lengthwise and XPO1 adopts a conformation that is an intermediate space between open and closed conformations [73]. The polyketide of LMB interacts hydrophobically with the same XPO1 groove residues that contact NES sequences. LMB analogs, anguinomycins (Figures 2c and d) display selective cytotoxicity against transformed cells at picomolar concentrations [65]. A truncated anguinomycin analog was still capable of blocking nuclear export [64]. Based on these findings, goniothalamin (Figure 2e) was identified as a nuclear export inhibitor [66]. A medicinal chemistry approach using a modified LMB yielded several semi-synthetic LMB derivatives which maintained the high potency of LMB, but were up to 16-fold less toxic than LMB *in vivo*[64]. The most potent derivative, KOS-2464 (Figure 2f), showed substantial efficacy in multiple mouse xenograft models, without affecting normal lung fibroblasts [64]. Furthermore, the treatment of several p53 wild-type cell lines with this potent derivative led to the up-regulation and nuclear localization of p53 [64]. These data suggest that toxicity associated with LMB may be linked to off-target effects and provides proof that nuclear export can be inhibited with manageable toxicities *in vivo*.

Anti-cancer/anti-fungal XPO1 inhibitors have been isolated from myxobacterium *Sorangium cellulosum* in a soil sample collected in Cala Ratjada (Mallorca, Spain) by Höfle, Reichenbach and others in 1995 [67]-[71]. Ratjadones (Figure 2g and h) have similar chemical structures to LMB and employ an identical molecular mechanism to inhibit XPO1 [67]-[69]. Cells treated with ratjadones manifest a significant increase in the size of their nuclei, further indicating an effective block of nuclear export [68]. Cell-cycle analysis of these cells showed that ratjadone compounds arrest the cells in G1 phase [70]. Synthetic ratjadone analog C (Figure 2h) inhibits nuclear export of topo IIα and sensitizes drug-resistant human multiple myeloma cells to the topo IIα inhibitors doxorubicin and etoposide when used at nanomolar concentrations [72]. Therefore, blocking XPO1 nuclear export may sensitize cancer cells either by preventing export of additional tumor suppressors or by preventing cell cycle progression. To date, ratjadones compounds have not been tested *in vivo*.

Daelemans et al. identified the synthetic small molecule PKF050-638 (Figure 2i) with a molecular mass of 292.7 kDa that reversibly disrupts XPO1-NES interaction in the micromolar range and demonstrates strict structural requirements for its activity [74]. Structural studies on PKF050-638 indicated XPO1 inhibition and highlighted that the activity of these compounds was not solely correlated to the targeted cysteine in XPO1. This suggests that more structural elements in the NES binding domain are involved [74].

Other natural compounds that bind to Cysteine 528 of XPO1were identified, including valtrate (Figure 2j) and acetoxychavicolacetate (ACA; Figure 2k) isolated from *Valeriana fauriei* and *Alpiniagalangal*, respectively [75],[76]. Although valtrate and ACA were developed as anti-viral compounds, they might be useful as anti-cancer agents.

An orally-active synthetic small molecule, CBS9106 (Figure 2l), which reversibly blocks XPO1-mediated nuclear export, is currently being developed as a preclinical anti-cancer agent [77]. Its mechanism of action remains to be fully elucidated. CBS9106 is able to reduce XPO1 protein levels without affecting its mRNA expression. This effect is reversed by adding bortezomib, suggesting that CBS9106-mediated XPO1 inhibition results in proteasome-dependent XPO1 degradation. XPO1 protein degradation mediated by CBS9106 encompasses are large portion of the inhibitory activity of this compound [77],[78]. In cells, CBS9106 caused a reversible arrest of the cell cycle and induced apoptosis in a time- and dose-dependent manner across a broad spectrum of cancer cells. Oral administration of CBS9106 suppressed tumor growth and prolonged survival in myeloma-bearing mice without causing significant weight loss [77]. A reduced level of XPO1 protein was also observed in tumor xenografts isolated from CBS-9106-treated mice. Toxicology studies will need to be completed in order to determine whether this promising pre-clinical candidate could be advanced to human clinical trials.

### Selective Inhibitors of Nuclear Export (SINE™)

The use of novel computational methods has recently facilitated the discovery of orally-bioavailable and highly-potent small molecules classified as Selective Inhibitors of Nuclear Export (SINE) [79]. The most advanced molecule in this series, selinexor (KPT-330; Figure 2m) is currently in Phase I/II clinical trials [80]-[89]. X-ray crystal structures of various SINE compounds were elucidated and indicate that they covalently bind to Cysteine 528 of XPO1 in a slowly-reversible and highly-selective manner (KPT-185; Figure 2n) [90],[91]. SINE compounds showed broad activity when tested in *in vitro* cytotoxicity experiments in hematological and solid tumor cell lines. In these experiments, the IC_50_ values ranged from 20 to 2000 nM with 95% of the cells tested having IC_50_ values below 500 nM. There was minimal toxicity to normal cells, indicating that the cytotoxicity of SINE compounds specifically targets malignant cells [90],[91].

SINE compounds were able to overcome the protective micro-environment effects in studies using multiple myeloma and chronic lymphocytic leukemia cell viability assays conducted in the presence of bone marrow stromal cells (BMSC) [90],[92]. The *in vivo* efficacy of SINE compounds was established in numerous pre-clinical murine xenograft, orthotopic, primagraft, and leukemograft models [26],[61],[90]-[102]. SINE compounds displayed single-agent activity and provided a statistically significant survival advantage in hematological malignancies, specifically in models of non-Hodgkin lymphoma, chronic lymphocytic leukemia, acute myeloid leukemia, acute lymphocytic leukemia, and multiple myeloma [26],[90]-[96]. Selinexor also demonstrated robust single-agent efficacy in solid tumour xenografts including kidney, pancreas, prostate, breast, lung, melanoma, colon, gastric, ovarian, neuroblastoma, and sarcomas [61],[97]-[105]. Marked synergy was observed when selinexor was used with a variety of chemotherapies and targeted therapies including platinum and taxanes [60], topoisomerase I and II inhibitors [53],[106], dexamethasone [87], cytarabine [107], proteasome inhibitors [53], and various tyrosine kinase inhibitors (TKIs) [108].

A structurally-related SINE compound, verdinexor (KPT-335; Figure 2o), is currently being developed for canine lymphoma [109]-[112]. Verdinexor has shown potent cytotoxic activity in canine NHL and melanoma cells when administered 2-3 times a week to companion dogs with spontaneously-occurring B- and T-cell lymphomas. Lymphomas are some of the most common malignancies in companion dogs and the diseases are characterized by rapid progression; dogs may live up to only a few weeks if left untreated [109]. In Phase I/II canine clinical trial in companion dogs with NHL (naïve or relapsed), verdinexor was orally administered at doses of 1.0 – 1.75 mg/kg and was generally very well-tolerated, with anorexia as the most common toxicity indicator [112]. In the Phase II study, verdinexor displayed single-agent activity with an overall objective response rate of 34% (20/58 dogs). This included 19 partial responses and one complete response (in a dog with T-cell lymphoma).

First human Phase I studies of selinexor in advanced hematological and solid malignancies were initiated as of June 2012 (clinicaltrials.gov). Selinexor was orally administered 2-3 times per week in doses between 3 and 85 mg/m^2^. Selinexor was rapidly absorbed and showed dose-proportional pharmacokinetics with no accumulation. Preliminary results suggest that selinexor is generally well-tolerated, with nausea, anorexia and fatigue being the primary side-effects. Side-effects were mostly Grade I and II in nature, reversible and manageable with supportive care. In line with the pre-clinical animal model results mentioned above, preliminary signals of efficacy were observed particularly with hematological malignancies including AML, CLL, NHL, and MM [80]-[82]. For solid malignancies, selinexor also displayed single-agent activity in prostate [88], ovarian [84],[85], cervical [84], and colorectal [83],[84] cancers and showed prolonged disease control in patients with head and neck cancer [89], as well as sarcoma [86]. Based on the encouraging results from the Phase I studies, several Phase II studies of selinexor have been initiated in patients with AML, GBM, melanoma, prostate, ovarian, cervical, and endometrial malignancies (a complete list of the on-going clinical trials is available at clinicaltrials.gov). Taken together, these results suggest that selinexor can be safely administered for prolonged periods to heavily pre-treated, relapsed and/or refractory patients with manageable side-effect profiles.

## Conclusions

Nuclear import and export is a highly-coordinated process involving numerous proteins and large complexes working in concert at the nuclear envelope. This process delicately balances cell growth and death mechanisms in cells. One of the central proteins in nuclear export is XPO1, or exportin-1. XPO1 mediates cell proliferation through several pathways: (i) the subcellular localization of NES cargoes, (ii) the control of the mitotic apparatus and chromosome segregation, and (iii) the maintenance of nuclear and chromosomal structures. Nuclear export through XPO1 is up-regulated in different cancer types and may be used as prognostic indicator. XPO1 dysregulation of intracellular localization of crucial suppressors and oncogenic proteins (p53, Rb, FOXO, p21, IκB, NFκB, and others) contributes to cancer development and progression. Because of this, XPO1 represents a pre-clinical and clinical target under active investigation.

To date, numerous small molecule NEIs have been developed. Selinexor represents the most advanced pharmacological agent currently being evaluated in Phase I/II human clinical trials in hematological and solid cancers. Although interfering with nuclear-cytoplasmic transport machinery could be detrimental to all active cells, SINE compounds have been shown to preferentially suppress or eliminate tumor cells and spare normal cells both in the pre-clinical and clinical setting. Continued evaluation of selinexor will establish the safety of targeting nuclear export through XPO1 and help pave the way for targeting other keystone cellular processes.

## Authors’ original submitted files for images

Below are the links to the authors’ original submitted files for images. Authors’ original file for figure 1 Authors’ original file for figure 2
