# Supplementary figures and images for: Nucleo-cytoplasmic transport as a therapeutic target of cancer
Source: J Hematol Oncol. 2014 Dec 5;7:85. doi: 10.1186/s13045-014-0085-1 (PMC4272779; doi:10.1186/s13045-014-0085-1)

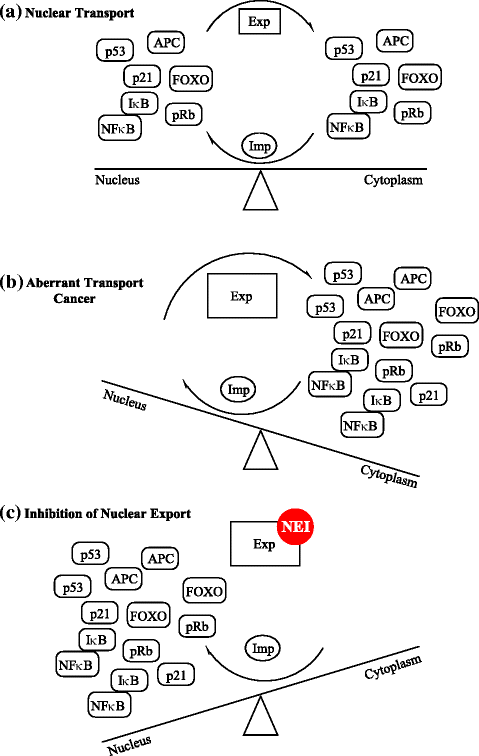

Supplement: Supplementary file 1 — Authors’ original file for figure 1 [file 13045_2014_85_MOESM1_ESM.gif]

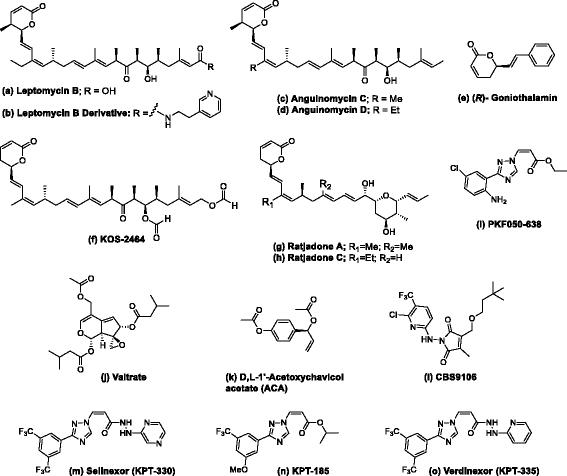

Supplement: Supplementary file 2 — Authors’ original file for figure 2 [file 13045_2014_85_MOESM2_ESM.gif]
